# All-optical control and visualization of ultrafast two-dimensional atomic motions in a single crystal of bismuth

**DOI:** 10.1038/ncomms3801

**Published:** 2013-11-18

**Authors:** H. Katsuki, J.C. Delagnes, K. Hosaka, K. Ishioka, H. Chiba, E.S. Zijlstra, M.E. Garcia, H. Takahashi, K. Watanabe, M. Kitajima, Y. Matsumoto, K.G. Nakamura, K. Ohmori

**Affiliations:** 1 Department of Photo-Molecular Science, Institute for Molecular Science, National Institutes of Natural Sciences, Myodaiji, Okazaki 444-8585, Japan; 2 The Graduate University for Advanced Studies (SOKENDAI), Myodaiji, Okazaki 444-8585, Japan; 3 Advanced Nano-Characterization Center, National Institute for Materials Science, Tsukuba 305-0047, Japan; 4 CREST, Japan Science and Technology Agency, Kawaguchi 332-0012, Japan; 5 Theoretical Physics and Center for Interdisciplinary Nanostructure Science and Technology (CINSaT), University of Kassel, Heinrich-Plett-Strasse 40, 34132 Kassel, Germany; 6 Materials and Structures Laboratory, Tokyo Institute of Technology, Yokohama 226-8503, Japan; 7 Department of Chemistry, Kyoto University, Kyoto 606-8502, Japan; 8 National Defense Academy of Japan, Yokosuka 239-8686, Japan; 9 Present address: Nara Institute of Science and Technology (NAIST), 8916-5 Takayama-cho, Ikoma 630-0192, Japan; 10 Present address: CELIA, CNRS-University of Bordeaux, 351 Cours de la Libération, F-33405 Talence cedex, France; 11 Present address: Tokyo Institute of Technology, 2-12-1 O-okayama, Meguro-ku, Tokyo 152-8550, Japan; 12 Present address: Tokyo Institute of Technology, Yokohama 226-8503, Japan; LxRay Co., Ltd., Koshien-2Bancho, Nishinomiya, Hyogo, 663-8172, Japan; University of Tsukuba, Ten-nodai 1-1-1, Tsukuba, Ibaraki, 305-8574, Japan

## Abstract

In a bulk solid, optical control of atomic motion provides a better understanding of its physical properties and functionalities. Such studies would benefit from active control and visualization of atomic motions in arbitrary directions, yet, so far, mostly only one-dimensional control has been shown. Here we demonstrate a novel method to optically control and visualize two-dimensional atomic motions in a bulk solid. We use a femtosecond laser pulse to coherently superpose two orthogonal atomic motions in crystalline bismuth. The relative amplitudes of those two motions are manipulated by modulating the intensity profile of the laser pulse, and these controlled motions are quantitatively visualized by density functional theory calculations. Our control-visualization scheme is based on the simple, robust and universal concept that in any physical system, two-dimensional particle motion is decomposed into two orthogonal one-dimensional motions, and thus it is applicable to a variety of condensed matter systems.

## Abstract (web summary)

Controlling the motion of atoms in solids with light allows for a deeper understanding of their fundamental properties, yet most studies only deal with one spatial dimension. Katsuki *et al.* extend this approach to two-dimensional control and use it to visualize atomic motion in bismuth.

## Graphical abstract

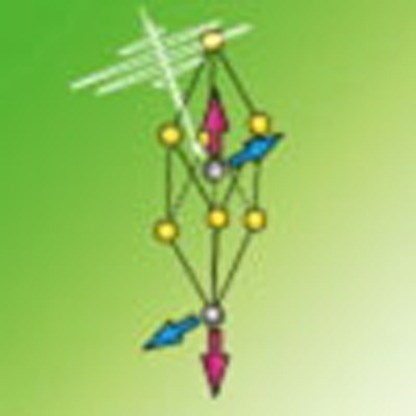

Atomic motions in the solid state can be triggered with optical pulses. If the pulse duration is shorter than the periods of lattice vibrations, the atoms start oscillating collectively. This collective oscillation is referred to as coherent phonons^1^,^2^. Coherent phonons have been observed in a wide variety of materials^3^,^4^,^5^,^6^,^7^,^8^,^9^,^10^,^11^,^12^,^13^,^14^,^15^,^16^ and have been controlled one-dimensionally with a train of multiple laser pulses^3^,^4^,^5^,^6^,^7^,^8^,^9^. One of these control experiments was demonstrated with two different phonon modes. The directions of atomic motions are, however, parallel between those two different modes, so that the control was one-dimensional^8^. Two-dimensional (2D) control has been performed with a pair of identical laser pulses with different polarizations applied to energetically degenerate orthogonal atomic motions^10^. Another approach is necessary to control orthogonal non-degenerate atomic motions, which is more common in most materials. Such 2D control should be important in photo-induced processes^17^,^18^ and could lead to further development of optically engineered functionality of bulk solids^19^,^20^.

Here we demonstrate 2D control of atomic motions in crystalline bismuth. It is known that superposing two chirped femtosecond laser pulses causes intensity modulation whose characteristic period lies within the terahertz (THz) region^21^,^22^. This method is applied to atomic motions in the current study. Two orthogonal phonon modes of A_1g_ and E_g_ symmetries^23^ are superposed with the modulated pulse and the transient relative reflectivity change of the crystal surface is measured with another femtosecond probe pulse. The reflectivity changes show beat structures whose temporal evolution changes drastically as we change the delay between the chirped femtosecond pulses on the attosecond timescale. We map these beat structures into a 2D space of atomic displacements with our density functional theory (DFT) calculations, demonstrating direct 2D control of atomic motion in each unit cell and its visualization.

## Results

### Coherent phonons in crystalline bismuth

**Figure 1:**
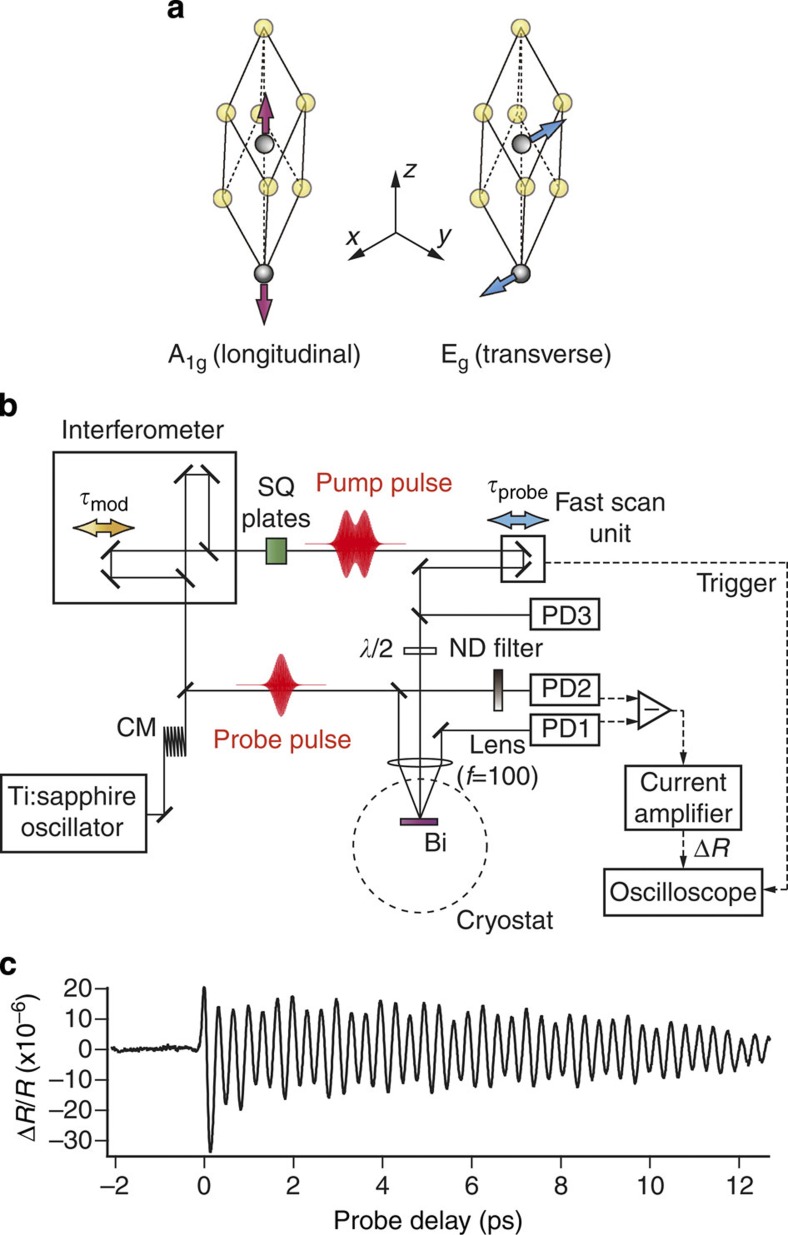
Scheme of the present experiment. (**a**) Crystal unit cell structure of Bi and the orientation of the A_1g_ (longitudinal) and E_g_ (transverse) phonon motions. (**b**) Experimental configuration for the modulated pump–probe reflectivity measurement. CM, chirp mirror; PD, photodiode; ND filter, neutral density filter; SQ plates, window plates of synthesized quartz substrate. (**c**) Oscillatory coherent phonon signal observed with *τ*_mod_ ~0 fs. The origin of the probe delay *τ*_probe_=0 ps is defined as the top of the first positive peak of the oscillatory phonon signal.

The crystal structure of bismuth is rhombohedral^24^ and its primitive unit cell consists of two atoms as shown in Fig. 1a. There exist three optical phonon modes with A_1g_ and doubly degenerate E_g_ symmetries at the Γ-point in the reciprocal lattice space^23^. According to our definition of the axes as given in Fig. 1a, the A_1g_ mode corresponds to the longitudinal motion and the E_g_ mode corresponds to the lateral motion of the Bi atoms.

### Experimental setup

Figure 1b schematically illustrates our pump–probe experiments performed on the (0001) surface of a single-crystal Bi. The crystal was cooled in a liquid helium cryostat to 5.2 K to reduce damping and dephasing of the coherent phonons. The output of a Ti:sapphire laser (centre wavelength: ~802 nm, bandwidth: ~34 nm) was split with a partial beam splitter into two beams with 9:1 ratio, which were used as pump and probe pulses, respectively. The pump pulse was input to our homemade highly stabilized Michelson-type interferometer^25^,^26^ to produce a phased pair of laser pulses. The intensity of each pulse was ~6 mW (fluence: ~2 μJ cm^−2^ on the sample). These pulses (pulse width: ~30 fs in their Fourier transform (FT) limits) were chirped with transmitting optics inserted on their optical path and are hereafter referred to as chirped subpulses (CSPs)^27^. The group delay dispersion (GDD) parameter of each CSP is hereafter referred to as *φ*′′. Those CSPs were temporally superposed with their delay *τ*_mod_ to produce a modulated pump pulse whose temporal intensity envelope *I*(*t*) shows periodical undulations. The FT of *I*(*t*) shows a single peak in the THz region where its centre frequency and peak width are functions of *τ*_mod_ and *φ*′′. A combination of these two degrees of freedom in the centre frequency and peak width allows for arbitrary tuning of the relative intensities of any two different frequency components in the THz region. Superposing CSPs thus serves as a practical technique for THz intensity modulation with near-infrared laser pulses. The train of femtosecond pulses has often been used in the previous experiments to control coherent phonons one-dimensionally^3^,^4^,^5^,^6^,^7^,^8^,^9^. The difference between such a pulse train and the current CSPs will be described later from the viewpoint of 2D control. The polarization of the modulated pump pulse was rotated by 90°, so that the pump and probe pulses were focused on the Bi sample with their polarizations perpendicular to each other. The reflectivity change Δ*R* of the probe pulse induced by the modulated pump pulse was monitored with a pair of balanced photodiodes (PD1 and PD2). The differential signal from the PDs was amplified with a current amplifier (SRS SR570) and averaged in a digital oscilloscope. The temporal evolution of Δ*R* was measured by scanning the delay *τ*_probe_ of the probe pulse from the first CSP repetitively at 20 Hz (APE Scan Delay) with a fixed *τ*_mod_. A bandpass filter from 3 to 300 kHz was used in all of the measurements reported in this study, so that the non-oscillatory background in the temporal evolution, which corresponds to the featureless electronic response, was filtered out. The intensity of the modulated pump pulse was monitored with a photodiode (PD3) at each *τ*_mod_. Other details of signal processing are given in the Methods section.

The temporal full width at half maximum (FWHM) of the probe pulse at the sample was measured to be ~50 fs, giving its GDD parameter *φ*′′~430 fs^2^. The FWHM and GDD parameter of the CSPs were estimated from the cross-correlation between one of the CSPs and the probe pulse to be ~260 fs and *φ*′′~2,600 fs^2^, respectively.

### Control of 2D atomic motions

Figure 1c shows an example of the temporal evolution Δ*R*(*τ*_probe_)/*R* with *τ*_probe_ scanned and *τ*_mod_ fixed to ~0 fs. The beat structure with a period of ~330 fs arises from coherent phonon motion and is described with a linear combination of two damped harmonic oscillators corresponding to A_1g_ (longitudinal) and E_g_ (transverse) phonons^23^:


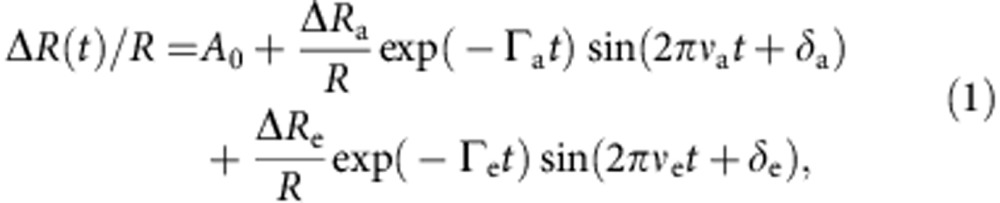


where *A*_0_ is the baseline, and Δ*R*_i_/*R*, Γ_i_, *ν*_i_ and *δ*_i_ with {i}={a, e} are the phonon amplitudes, damping factors, oscillation frequencies and initial phases of A_1g_ and E_g_ phonons, respectively.

Assuming each CSP has a Gaussian spectral profile with the GDD parameter *φ*′′, the spectrum of the electric field is described as^28^,


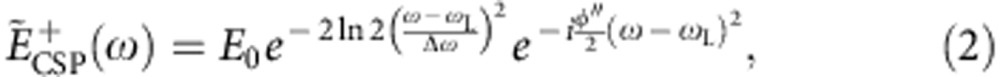


where *ω*_L_ is the central angular frequency of the pulse and Δ*ω* is its spectral FWHM. The modulated pump pulse is the superposition of two CSPs with their interpulse delay *τ*_mod_, so that its intensity envelope *I*(*t*) is


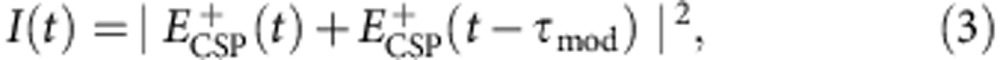


where 
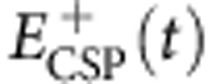
 is given by


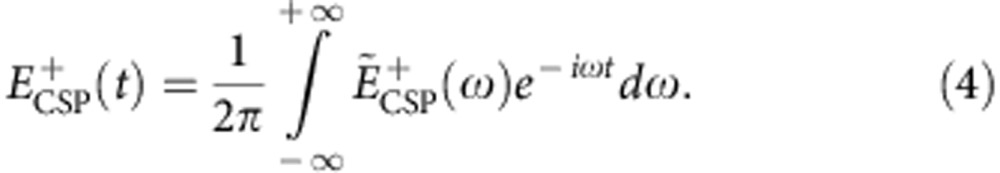


The envelope *I*(*t*) is Fourier-transformed to give its THz spectrum 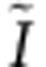
(*ω*) as


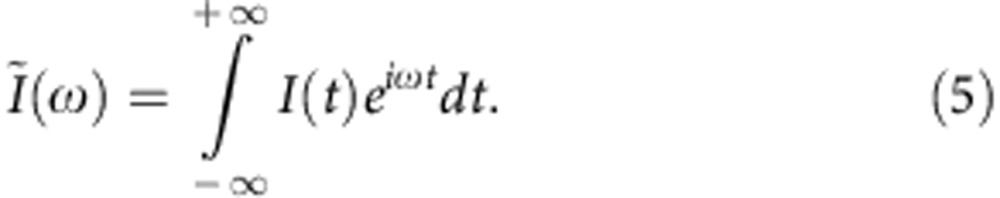


**Figure 2:**
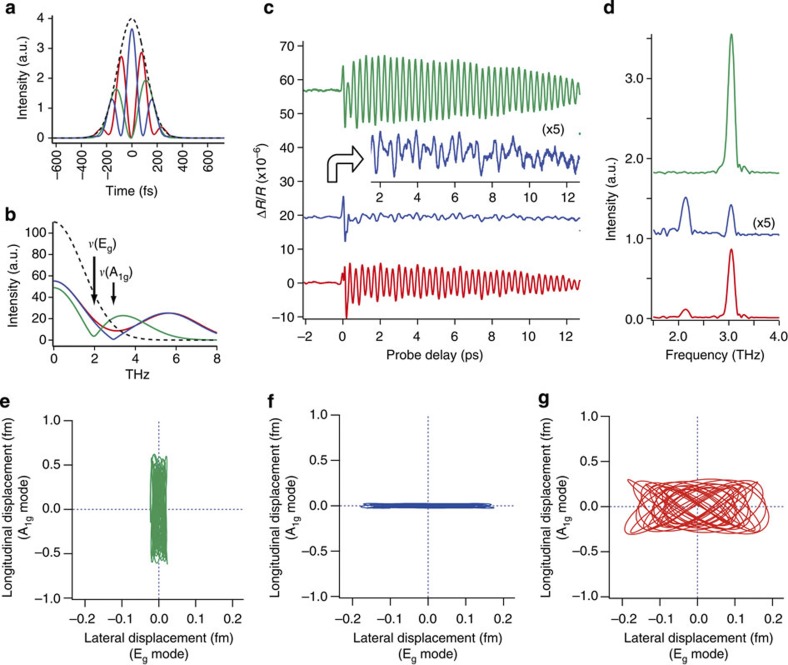
Temporal evolution of coherent phonons and 2D atomic motions controlled with the CSP technique. (**a**) Simulated temporal pulse envelopes for three different CSP delays. Green: *τ*_mod_=49.4 fs. Blue: *τ*_mod_=92.2 fs. Red: *τ*_mod_=93.6 fs. The dotted curve is the temporal intensity of CSP pair with *τ*_mod_=0 fs. The pulse parameters are 2*πc*/*ω*_L_=802 nm (*c* is the speed of light in air), Δ*ω*/2*π*=15.7 THz (corresponding to 34 nm FWHM), and *φ*′′=2,600 fs^2^, respectively. (**b**) Fourier-transformed THz intensity spectra calculated from the temporal pulse envelopes of **a**. (**c**) Observed coherent phonon signals for three different *τ*_mod_ similar to those for the simulations shown in **a** and **b**. Inset: 5 × expanded view of the blue trace. The origin of the probe delay *τ*_probe_=0 ps is defined as the top of the first positive peak of the green trace. (**d**) Fourier-transformed THz spectra of the coherent phonon signals given in **c**. The blue trace is multiplied by 5. The peak at ~2.1 THz corresponds to the E_g_ motion and the peak at ~3.0 THz corresponds to the A_1g_ motion. (**e**–**g**) Traces of the atomic motions within a unit cell converted, respectively, from the green, blue and red phonon signals shown in **c**. Each trace represents the trajectory within the time window from *τ*_probe_=0.82 to 10.48 ps. Note the scaling difference between the lateral and longitudinal axes.

Figure 2a shows the intensity envelopes *I*(*t*) of the modulated pump pulse simulated with three different CSP delays *τ*_mod_=49.4, 92.2 and 93.6 fs combined with the CSP parameters *ω*_L_, Δ*ω* and *φ*′′, taken from the current experimental conditions. The dotted curve represents the intensity envelope of a CSP pair with the delay *τ*_mod_=0 fs. Figure 2b shows the THz spectra 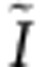
(*ω*) Fourier-transformed from those simulated envelopes *I*(*t*), demonstrating that the spectrum 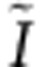
(*ω*) is modulated by changing the CSP delay *τ*_mod_, so that we can control the relative intensities of two different THz components resonant with A_1g_ and E_g_ phonons, respectively.

Figure 2c shows the observed temporal evolutions Δ*R*(*τ*_probe_)/*R* with three different CSP delays *τ*_mod_ similar to those for the simulations shown in Fig. 2a,b. It is clearly seen that the beat structure changes drastically as we change the CSP delay. The FTs of the traces in Fig. 2c are plotted in Fig. 2d in which two peaks at ~3.0 and ~2.1 THz correspond to the A_1g_ and E_g_ phonons, respectively, demonstrating that their relative intensities are clearly controlled.

### Visualization of 2D atomic motions

The real-space atomic motions have been reconstructed in Fig. 2e–g from the measured beats shown in Fig. 2c, based on the correspondence between the nuclear displacement^29^,^30^,^31^ and the reflectivity change Δ*R*. This correspondence has been obtained by linear response DFT calculations^32^ with the full-potential linearized augmented plane wave programme WIEN2k^33^,^34^, which is among the most accurate DFT codes presently available. The two quantities that we have computed are the potential energy surface as a function of the absorbed laser energy and optical properties in dependence of the Bi atomic coordinates. Details of our total-energy computations have been published in ref. 35. For the optical calculations, we have used the module described in refs 36 and 37, which is based on the Lindhard equation. With this package, we computed the imaginary part of the dielectric tensor for photon energies up to 27 eV. We obtained the real part using the Kramers–Kronig relation, where we added an imaginary part of 0.1 eV to the energy to account for the finite lifetime of excited states. It is important to realize that both our total-energy computations and the optical calculations rely on the interpretation of the Kohn–Sham energies as single-electron excitation energies^34^,^36^,^37^, which, although not strictly valid within DFT, is widely used and appears to work well for many materials including bismuth^34^,^37^. As the optical calculations are very sensitive to the quality of the **k** mesh, in this part of our theoretical work we have included 40 × 40 × 40 **k** points.

**Figure 3:**
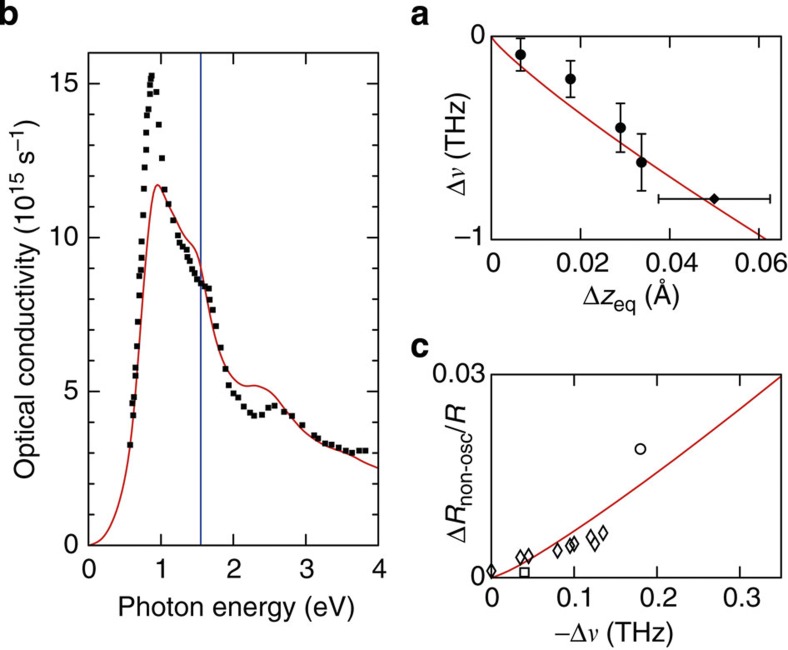
Comparison between theoretical results and published experimental data. The red lines show our calculated results. The symbols reproduce published experimental data. (**a**) Relation between the softening of the A_1g_ phonon mode and the change in the quasiequilibrium coordinate *z*_eq_ of the Bi atoms. (**b**) Optical conductivity as a function of photon energy. The blue vertical line indicates the photon energy of 1.55 eV (800 nm) used in the present experiments. (**c**) Relative change in the non-oscillatory (non-osc; or background) component of the reflectivity at 800 nm as a function of the phonon softening. The experimental data points and their error bars were taken from the following publications: filled circles, ref. 30; filled diamonds, ref. 29; filled squares, ref. 38; open square, ref. 39; open diamonds, ref. 4; and open circle, ref. 40.

To check the validity of our approach, in Fig. 3 we have plotted a number of calculated dependencies and we have compared our predictions to published experimental data. In particular, the quasiequilibrium coordinate *z*_eq_, which is associated with the A_1g_ phonons, changes with increasing pump–laser fluence. Simultaneously, the A_1g_ phonon frequency softens. In Fig. 3a, we have plotted the functional dependence of both effects, which can also be experimentally assessed through time-resolved X-ray spectroscopy^29^,^30^. Clearly, the agreement with these experiments is very good. In Fig. 3b, we show the computed optical conductivity for the electronic ground state. It qualitatively reproduces the essential features of the measured data of ref. 38, in particular it has a maximum around 0.95 eV, a shoulder at 1.5 eV, a dip at 2.1 eV, followed by a maximum and a decay. At the photon energy of 1.55 eV (800 nm), to which we shall restrict ourselves in the following, the computed optical conductivity is in good quantitative agreement with the data of ref. 38. As a final check, in Fig. 3c, we show how the average reflectivity (sometimes called background reflectivity, because it ignores the superimposed oscillatory effect due to excited coherent phonons) and the A_1g_ phonon frequency change simultaneously. Our prediction of Fig. 3c is compared with optical measurements of refs 4, 39, 40. The good agreement between our calculations and the experimental data confirms that our approach reliably reproduces the experimental results.

**Figure 4:**
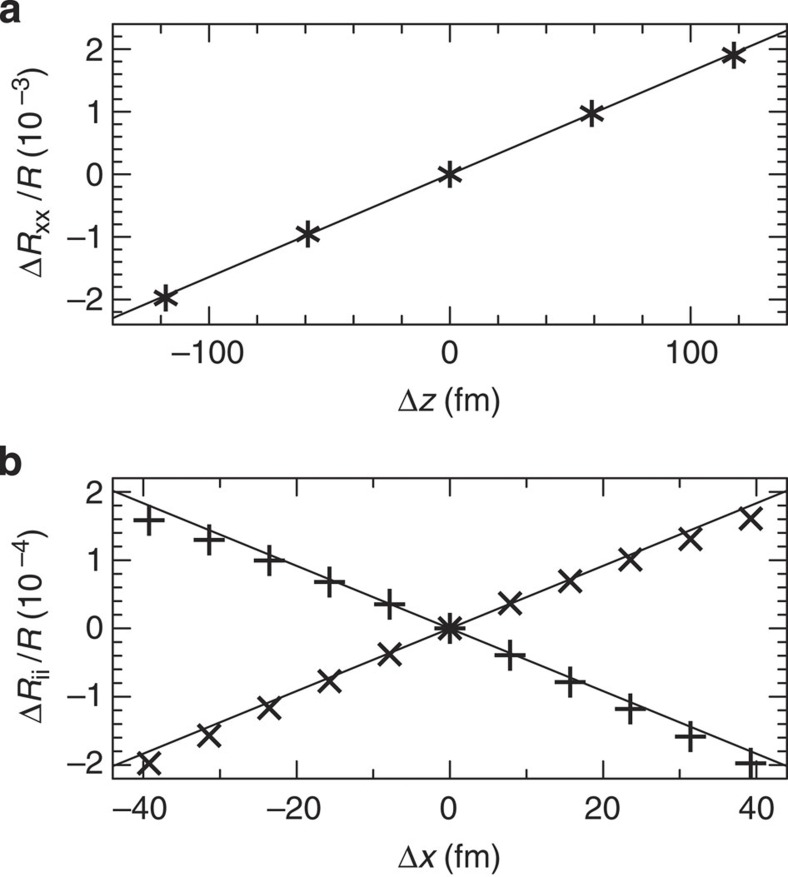
Calculated reflectivity as a function of nuclear displacement. (**a**) Change in the reflectivity *R*_xx_ at 800 nm as a function of the nuclear displacement in the *z* coordinate. The change in *R*_yy_ is equal. (**b**) Change in the reflectivity components *R*_xx_ (crosses) and *R*_yy_ (pluses) as a function of the nuclear displacement in the *x* coordinate. Solid lines represent linear fits to the computed data points.

We now turn to a description of our computed results for the optical reflectivity *R* of Bi at a wavelength of 800 nm. For the ground state (*z*_eq_=0.2344*c*), where *c* is the lattice parameter, we have obtained *R*=(*R*_xx_+*R*_yy_+*R*_zz_)/3=74.4%, in perfect agreement with the experimental value of 74.3% (ref. 39). In Fig. 4a, we show the relative change in the reflectivity *R*_xx_ for small displacements of the atoms in the *z* direction. For comparison, a value of Δ*z*=18.4 pm would undo the Peierls distortion in Bi. The calculated reflectivity change shows a clear dependence on the displacement in the *z* direction with a fitted slope of ∂(Δ*R*/*R*)/∂*z*=0.0164 per pm. Moving the Bi nuclei in the *x* direction changes the reflectivity tensor anisotropically with a slope of ±0.0046 per pm, as shown in Fig. 4b.

Utilizing these parameters, we have converted our measured beats Δ*R*(*τ*_probe_)/*R* shown in Fig. 2c to the real-time atomic motions in the *z* and *x* directions. Details of the conversion process are described in the Methods section. Figure 2e–g shows those atomic motions in the Bi unit cell converted from the observed beats shown in Fig. 2c. In this conversion, the observed beats between *τ*_probe_=0.82 and 10.48 ps are fitted with the oscillatory function in equation (1), giving the amplitude parameters, Δ*R*_a_/*R* and Δ*R*_e_/*R*, transformed to the absolute displacement of the Bi atom. Details of this fitting procedure and the relevant parameters are given in the Methods section. It is clearly seen from Fig. 2e–g that the ultrafast 2D atomic motions in crystalline bismuth have been actively controlled by shaping the pump pulse.

### Comparison between CSP and pulse-train techniques

**Figure 5:**
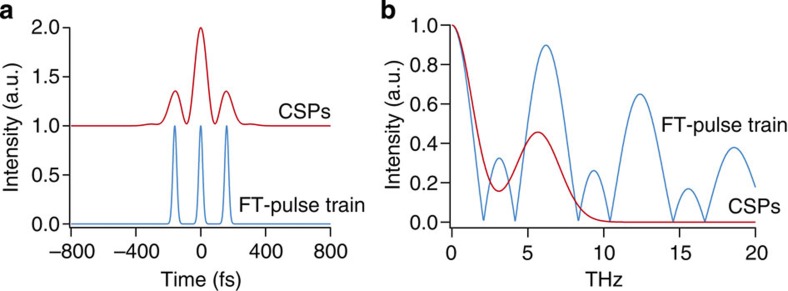
Comparison of the CSP and the pulse-train techniques. (**a**) Simulated temporal pulse envelopes for the CSPs and a pulse train of three FT pulses. Temporal separation of the pulse train is 160 fs and *τ*_mod_ for the CSPs is 93.6 fs. The pulse parameters of 2*πc*/*ω*_L_=802 nm (*c* is the speed of light in air), Δ*ω*/2*π*=15.7 THz (corresponding to 34 nm FWHM) are common for both cases, and *φ*′′=2,600 fs^2^ for CSPs and 0 fs^2^ for the pulse train, respectively. (**b**) The Fourier-transformed THz intensity spectra calculated from **a**.

The train of femtosecond pulses has often been used in the previous experiments to control coherent phonons^3^,^4^,^5^,^6^,^8^. It should be interesting to understand the difference between those pulse-train techniques and the current CSP technique from the viewpoint of THz intensity modulation. Figure 5a shows the intensity envelopes *I*(*t*) of the two superposed CSPs and the train of FT-limited pulses with their similar peak-to-peak intervals. Figure 5b shows the THz spectra 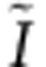
(*ω*) Fourier-transformed from the envelopes *I*(*t*) shown in Fig. 5a. The clear difference is seen in the high-frequency region above 10 THz, where the second and third harmonics of the lowest frequency component around 6 THz are filtered out in the CSP case. The CSP technique thus allows for more flexible tuning of the relative intensities of different frequency components in the THz region than the conventional pulse-train approach, making CSPs ideal for controlling ultrafast 2D atomic motions in bulk solids. Spatial light modulators or acousto-optic modulators could be other candidates to produce temporal profiles similar to the one of the CSPs.

## Discussion

The intensity of the E_g_ mode in Fig. 2d should be correlated with the THz intensity at its resonance frequency, because the E_g_ mode is excited directly by the pump pulse^1^,^2^,^15^,^16^. The coupling between the A_1g_ and E_g_ phonons, which has been discussed in ref. 34, is of third order in the atomic displacements and can be safely neglected at the low fluence in the present experiments.

The A_1g_ phonons originate from a laser-induced shift of the potential energy surface minimum in the *z* direction in Fig. 1a (displacive excitation of coherent phonons (DECP) model)^11^, whereas the E_g_ phonons originate from impulsive stimulated Raman scattering (ISRS model)^41^. When irradiated with a FT-limited laser pulse, the DECP mechanism launches coherent phonons that are cosine-like with an initial phase of zero degree, whereas the ISRS launches sine-like ones that are phase-shifted by 90° (ref. 11). The present initial phases of the A_1g_ and E_g_ phonons are determined by a combination of their generation mechanisms (DECP and ISRS, respectively) and the intensity envelope *I*(*t*) of our modulated pump pulse. The effect of an intensity envelope on the initial phase of coherent phonons has been recently discussed by Shimada *et al.*^9^. The trajectory starting with these initial phases will eventually reach any point in the 2D configuration space accessible with a given ratio of A_1g_ and E_g_ amplitudes as long as the phonon lifetime is infinite. However, the lifetime is finite, so that there could be points that are not reached by this trajectory. It is important to note that this trajectory can be modified to reach an arbitrary point in that accessible configuration space if the initial phases of those two phonon modes are actively controlled independently. This control could be implemented by tuning the chirp rates of the two CSPs independently with a dispersive optic inserted in each of the two optical paths of the interferometer. This additional control should be useful when the phonon lifetime is short, and the trajectory needs to be driven to a particular atomic configuration that is effective in inducing a specific dynamical process such as a phase transition.

It should be also noted, however, that the relative phase of A_1g_ and E_g_ modes recurs to its initial value every 1.1 ps, which is shorter than the lifetimes of those phonons by a factor of ~10 in the present case. Each of the controlled trajectories shown in Fig. 2e–g thus covers almost all possible trajectories within the possible 2D space given by each relative amplitude of those two phonon modes. The current amplitude control, therefore, allows for almost full control of 2D atomic motions.

The present combination of the ultrafast optical measurements and the DFT calculation of optical response as a function of atomic displacements offers a new method to visualize ultrafast structural changes in solids, all-optical alternative to time-resolved X-ray and electron diffraction techniques^30^,^42^.

Our control-visualization scheme is based on the simple, robust and universal concept that in any physical system, 2D motion of a particle is determined by the projections of its pathway onto two orthogonal axes. The scheme is thus applicable to a variety of condensed matter systems.

## Methods

### Data processing

The reflectivity change obtained as the differential signal of the two balanced PDs was first amplified by a current amplifier and then transferred to a digital oscilloscope at each pump–probe delay *τ*_probe_ scanned with the fast scan mechanical stage. The displacement of this stage was proportional to the voltage applied, and the voltage was swept sinusoidally in each scan. This temporal evolution of the voltage was associated with electric noises and was fitted with a sine function to calibrate *τ*_probe_. The data points thus sampled along the *τ*_probe_ axis were not equidistantly distributed. We interpolated these measured data points with cubic spline functions to give equidistant points to be fast-Fourier-transformed with the Hanning window function and zero padding. The temporal evolutions of the reflectivity change shown in this study are composed of these interpolated data.

The calibration of the reflectivity change signal was performed as follows. The phonon signal accumulated by the digital oscilloscope was transferred to the personal computer with a 16-bit integer format. To convert these integer-formatted data to the voltage unit, we independently performed a reference signal acquisition of a sine curve generated by a function generator as an input source. The range of the oscilloscope was set to the same scale as we have used in the phonon measurement, and data were saved in both the raw voltage format and 16-bit integer format. Each data was fitted with a sine function, and by comparing the fitted amplitude parameter of each data we obtain the proportionality parameter to transform the integer data to voltage unit. Using this parameter, the phonon signal in 16-bit integer format was rescaled to the voltage unit and then further transformed to the current unit according to the current amplifier setting. This value was divided by the recorded current value of each PD to give the reflectivity change data as plotted in the main text.

### Conversion process from phonon signals to 2D atomic motions

The coherent phonon signals obtained with three different *τ*_mod_ timings as shown in Fig. 2c were fitted with the model function equation (1) to give the amplitude of each of the A_1g_ (longitudinal) and E_g_ (transverse) phonon modes. Part of the phonon signal within the time window from *τ*_probe_=0.82 to 10.48 ps was used for the fitting procedure.

The frequency and decay rate of each phonon mode was determined as follows. First, the green trace of Fig. 2c was fitted by equation (1), neglecting the contribution of E_g_ mode. Next, using the parameters Γ_a_ and *ν*_a_ fixed to the value determined by this process, we fitted the blue and red traces of Fig. 2c. The parameters Γ_e_ and *ν*_e_ obtained from the blue and red traces were averaged to give the final value of Γ_e_ and *ν*_e_. Finally, we performed the fitting procedure again with these four parameters fixed to determine other parameters. Supplementary Table S1 summarizes the resulting fitting parameters for each phonon signal. The experimental phonon signals and the best-fitting results are shown in Supplementary Fig. S1. The amplitude parameters, Δ*R*_a_/*R* and Δ*R*_e_/*R*, were then converted to the displacements of the Bi atom in the *z* and *x* directions by using the coefficient ∂(Δ*R*/*R*)/∂*z*=0.0164 per pm and ∂(Δ*R*/*R*)/∂*x*=0.0046 per pm. These 2D displacements are plotted in Fig. 2e–g.

## Author contributions

H.K., M.K., K.G.N. and K.O. designed the experiment. H.K., H.C., K.I., H.T. and K.O. constructed the apparatus. H.K., J.C.D., K.H., K.I., H.C. and H.T. made the measurements. H.K., J.C.D., K.H. and K.I. analysed the data. All authors discussed the results. E.S.Z. and M.E.G. performed the DFT calculations to convert the reflectivity changes to the atomic displacements. H.K., E.S.Z., M.E.G. and K.O. wrote the manuscript, which was polished by M.K. and K.G.N. The project was conceived and supervised by K.O.

## Additional information

**How to cite this article:** Katsuki, H. *et al.* All-optical control and visualization of ultrafast two-dimensional atomic motions in a single crystal of bismuth. *Nat. Commun.* 4:2801 doi: 10.1038/ncomms3801 (2013).

## Supplementary Material

Supplementary Information Supplementary Figure S1 and Supplementary Table S1
