# Supplementary material for: All-optical control and visualization of ultrafast two-dimensional atomic motions in a single crystal of bismuth
Source: Nat Commun. 2013 Nov 18;4:2801. doi: 10.1038/ncomms3801 (PMC3868158; doi:10.1038/ncomms3801)
Supplement: Supplementary Information — Supplementary Figure S1 and Supplementary Table S1 [file ncomms3801-s1.pdf]

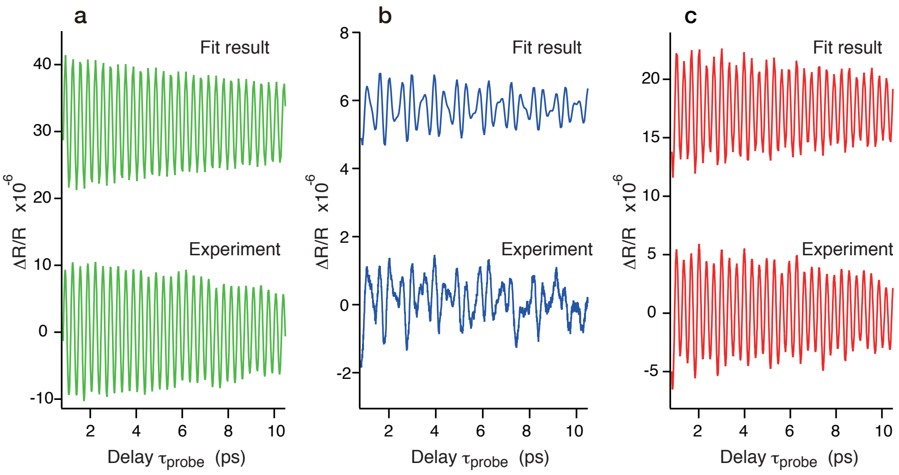

Supplementary Figure S1: (**a** - **c**)The experimental phonon signal and fitted result for each double pulse delay used in Fig. 2c of the main text. Fitted results are vertically shifted for clarity.

parameter	trace A	trace B	trace C
A_0	1.236e-7	1.312e-7	1.411e-7
$\Delta R_a/R$	1.076e-5	4.575e-7	5.298e-6
Γ_a (ps^{-1})	0.0547	0.0547	0.0547
ν_a (THz)	3.051	3.051	3.051
δ_a (rad.)	2.831	1.308	0.617
$\Delta R_e/R$	-1.158e-7	-8.905e-7	9.684e-7
Γ_e (ps^{-1})	0.0915	0.0915	0.0915
ν_e (THz)	2.138	2.138	2.138
δ_e (rad.)	2.817	2.103	5.156

Supplementary Table S1: List of fitting parameters given in eq. (1) for coherent phonon signals shown in Fig. 2c of the main text.
